# Early Continence Recovery after Preservation of Maximal Urethral Length until the Level of Verumontanum during Radical Prostatectomy: Primary Oncological and Functional Outcomes after 1 Year of Follow-Up

**DOI:** 10.1155/2013/426208

**Published:** 2013-09-19

**Authors:** Stavros Sfoungaristos, Stavros Kontogiannis, Petros Perimenis

**Affiliations:** Urology Department, Patras University Hospital, Building A, 4th Floor, Rion, 26500 Patras, Greece

## Abstract

*Purpose*. To investigate the effect of preventing maximal urethral length until the level of verumontanum during radical prostatectomy on both oncologic and functional outcomes. *Patients and Methods*. We recruited 329 patients, and they underwent an open radical prostatectomy by a single surgeon. The study cohort was randomized in 2 groups. A standard radical prostatectomy was performed in group A patients, while in group B the urethra was preserved until the level of verumontanum. *Results*. There was no statistically significant difference between the study groups in terms of positive surgical margins or biochemical relapse. Regarding the functional results, the incidence of incontinence, urgency, and nocturia at 1st month, statistically significant higher rates were seen in group A. In addition, there was a statistically significant difference in the number of pads/day in favor of group B at the 1st, 3rd, and 6th months after surgery. However, this difference was eliminated at 12 months postoperatively. Similar results were seen with the scores of the ICIQ-SF and IIQ-SF questionnaires. *Conclusions*. Without compromising the oncological outcome, our surgical modificated technique showed earlier recovery of continence in the first 6 months, having though the same rates of continence at 12 months.

## Introduction

Radical prostatectomy is mostly recommended for patients with localized disease and a long life expectancy [1]. The optimal outcome after radical prostatectomy is the so-called trifecta target, which consists of cancer control, along with the preservation of continence and erectile function with the latter to be achieved through the preservation of the neurovascular bundle [2]. Concerning the postoperative incontinence, several studies have been conducted to describe surgical techniques aiming to achieve better continence rates. 

Continence rates 1 year after radical prostatectomy are excellent in large series [3]. However, the achievement of an earlier continence at 3 or 6 months postoperatively is still a challenge, and several surgical techniques have been described in the literature recently attempting to achieve this [4–6]. Most techniques emphasize the importance of restoring the “normal” pelvic anatomy after removal of the prostate gland [7]. Preserving continence earlier postoperatively is of great importance, since it may have a positive psychosocial impact on patients undergoing radical prostatectomy, by improving their quality of life [7–9].

In an effort to achieve earlier continence rehabilitation, we conducted a prospective randomized controlled study, evaluating whether our technique of preserving maximal urethral length until the level of verumontanum at the time of apical dissection during radical prostatectomy achieves better functional outcomes, concerning the postoperative continence and the rate of nocturia and urgency, without though compromising the oncological result.

## 2. Patients and Methods

After we obtained an ethics committee approval from our institution, we conducted a prospective analysis of 329 men with prostate cancer who underwent an open radical prostatectomy between January 2008 and April 2012. All patients signed an informed consent, and all procedures were made by a single surgeon. The study cohort was divided into 2 groups by coin flip. In the first group of patients (group A), a standard radical prostatectomy was performed, as it has been described by Walsh and Donker [10], while in the second group (group B) a surgical modification was performed by preserving the verumontanum, as it is described below.

The primary endpoint of the study was firstly to compare the oncological results between the groups, in terms of positive surgical margins, and to justify the impact of the modified technique on functional outcomes, regarding the postoperative continence and the rate of nocturia and urgency.

All the patients were suffering from clinically localized prostate cancer defined by digital rectal examination and transrectal ultrasound. The diagnosis of the disease was established by positive biopsy results. Patients with any clinical suspicion of locally advanced prostate cancer were excluded from the study. After a detailed clinical history, patients with preoperative incontinence, nocturia, and/or urgency were excluded as well.

The surgical specimen after radical prostatectomy was examined by our institution pathologists, and a histological report concerning the presence of organ-confined disease, the presence of positive surgical margins, and the pathological grade and stage was obtained. Any extension of tumor outside of the prostatic capsule in the periprostatic fat was considered as advanced disease, while the infiltration of the capsule without penetration was considered as localized disease. The 2009 tumour node metastasis (TNM) classification was used to define the pathological stage.

Follow-up protocol was consisted by visits at 1st, 3rd, 6th, and 12th months postoperatively, and continence was evaluated by the number of pads used daily. In order to quantify the postoperative incontinence and to evaluate the impact of incontinence on quality of life, all patients completed the international consultation on incontinence questionnaire-short form (ICIQ-SF) and incontinence impact questionnaire-short form (IIQ-SF). PSA was calculated in all patients during each follow-up visit. Biochemical relapse was defined as 2 consecutive PSA values >0.2 ng/mL, 2 weeks apart.

### 2.1. Description of the Technique

A standard radical retropubic prostatectomy was performed in patients of group A. The patient was placed in the supine position. A 16 F Foley was inserted, and a supraumbilical midline incision was made. The rectus muscles were separated in the midline, and the transversalis fascia was opened sharply to expose Retzius space. The lymph node dissection was performed at this point if indicated. After the endopelvic fascia had been opened, the division of the puboprostatic ligaments was followed, visualizing the Santorini plexus. We adopted the technique described by Kessler et al. to ligate this venous plexus [11]. A curved Babcock Clutch was used to capture the Santorini plexus. Between the Santorini plexus and the prostate apex a ligature was passed as well as at the base of the prostate. The ligated Santorini plexus is sharply transected not at the level of the apex but over the lower half of the prostate. Thereafter the plain between the ligated Santorini plexus and the prostatic capsule was sharply dissected towards the apex. At the level of the apex, the external rhabdosphincter was progressively transected approximately 2 to 3 millimeters away from the apex. Once the catheter was reached, the urethra was transected on both lateral sides, and the catheter was grasped with a clamp. The catheter was transected as well as the posterior portion of the membranous urethra. Thereafter, the lateral and posterior parts of the prostate were dissected, and, afterwards, the dissection of the vas deferens and removal of the seminal vesicles were performed in both sides. The bladder neck was dissected and reconstructed forming a tennis racket closure by having inverted the bladder mucosa layer from 11 to 1 o'clock. A silicon 18 F Foley catheter was placed, and 6 sutures of the vesicourethral anastomosis were placed at 1, 3, 5, 7, 9, and 11 o'clock positions and tied without tension. A suction drain was placed, and the incision was closed.

The whole procedure followed exactly the same steps for patients of group B. However, at the time of apical dissection, the incision of the urethra was made very close to the prostate at the anatomical level proximal to the verumontanum, increasing the urethra length left behind at the surgical bed. Actually, the incision was not made 2 to 3 millimeters from the prostatic apex but just at the apex. After the dissection of the Foley catheter, the dissection of the posterior part of the urethra takes place just proximally to the verumontanum, in order to preserve the latter in place.

### 2.2. Statistical Analysis

Statistical analysis was performed by using SPSS version 17 (SPSS Inc., Chicago, IL, USA). Descriptive statistics are presented as the mean ± standard deviation and interquartile range for continuous variables and as the absolute and percent frequency for categorical variables. The numerical variables normality condition was studied by using Kolmogorov-Smirnov test. None of the variables had a normal distribution, and thus Mann-Whitney *U* test was used to compare means between groups. Chi-square *χ* ^2^ test was used for categorical variables. All tests were 2 tailed with *P* < 0.05 being considered as a statistically significant value.

## 3. Results

**Figure 1:**
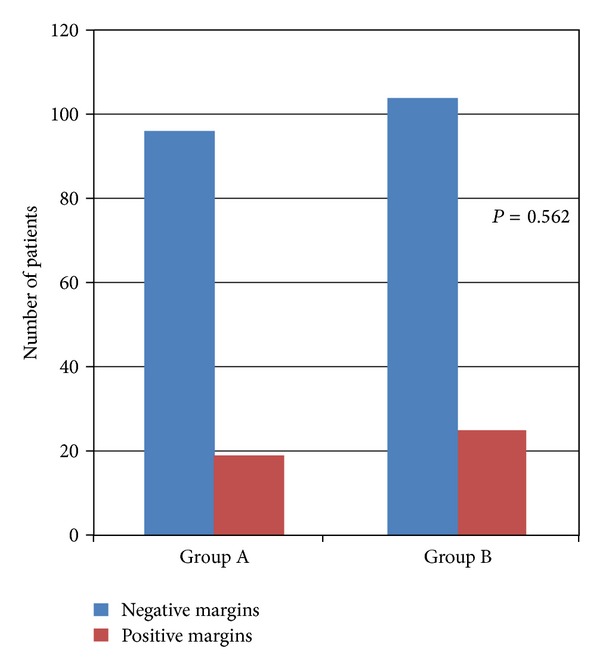
Positive surgical margins.

Patients with preoperative incontinence (*n* = 13), nocturia (*n* = 39), and/or urgency (*n* = 22) were excluded, while 19 patients with clinical evidence of locally advanced disease, based on the preoperative transrectal ultrasound and/or digital rectal examination, were excluded as well. Finally, 244 patients were included in the analysis, and 115 patients entered in group A and 129 in group B. The clinical and pathological characteristics of the study cohort are seen in Table 1. A nerve-sparing technique was performed in 141 patients (57.8%). Of them, 63 patients (44.7%) were belonging to group A and 78 (55.3%) to group B. As we can see in Table 2, there was no significant difference between the groups, regarding the postoperative stage (*P* = 0.433) and grade (*P* = 0.871). Extracapsular disease was found in 36 patients of group A and 31 patients of group B (*P* = 0.204), while 19 patients of group A and 25 of group B had positive surgical margins (Figure 1) showing no statistically significant difference (*P* = 0.562). Similarly, there was no difference in the rate of biochemical failure after 1 year of follow-up (*P* = 0.321) with 20 patients of group A and 19 of group B to relapse after surgery (Table 2).

**Figure 2:**
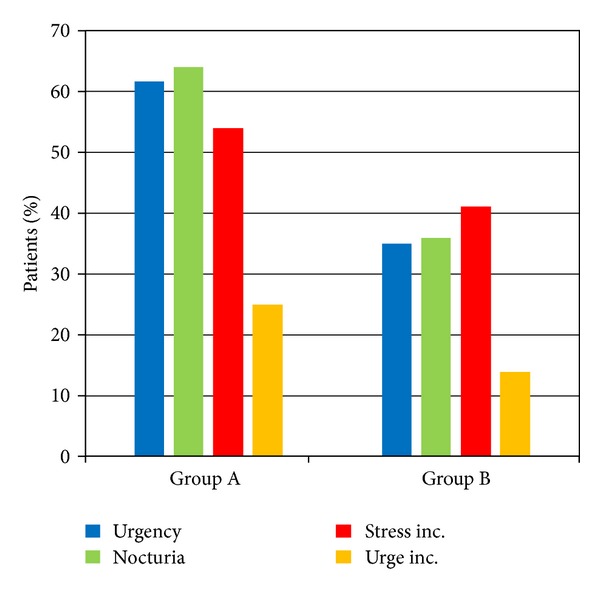
Rate of urgency, nocturia, stress, and urge incontinence 1 month after surgery.

**Figure 3:**
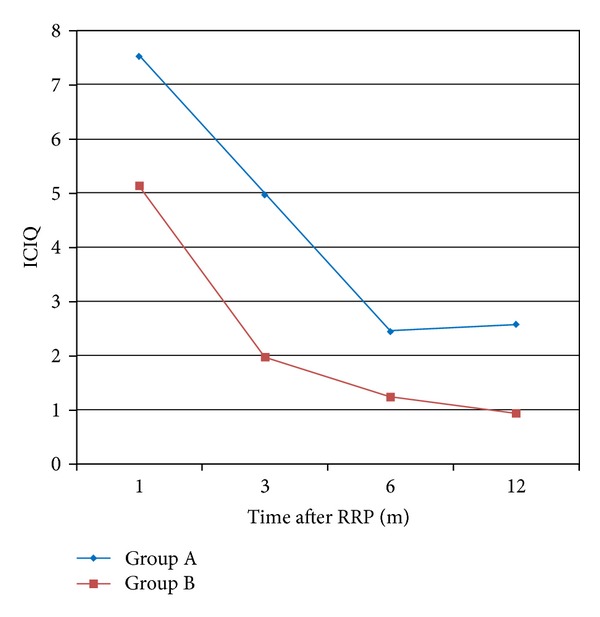
ICIQ-SF questionnaire.

**Figure 4:**
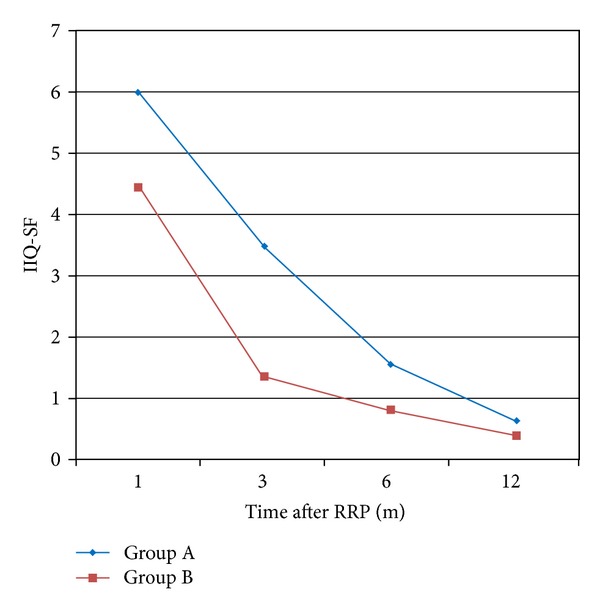
IIQ-SF questionnaire.

The overall incidence of urge incontinence at the end of the study period (1 year) was 10.4% and 8.53% for groups A and B, respectively, with no statistically significant difference between the groups (*P* = 0.611). Similarly, the rates of stress incontinence were not significantly different between groups (*P* = 0.548), with 20.9% of patients in group A and 17.8% of group B to report the symptom. Regarding the incidence of incontinence at the 1st month postoperatively, we noticed that there was a statistically significant difference either for cases with stress (*P* = 0.045) or with urge incontinence (*P* = 0.026) in favor of group B. Similarly, a significant increased number of patients within group A suffered from urgency (*P* < 0.001) and nocturia (*P* < 0.001) 1 month postoperatively (Figure 2). The above information was collected after obtaining a clinical history of each patient in the follow-up visits. Regarding the objective findings of postoperative incontinence, we found that there was a significant difference in favor of group B in the number of pads used per day (Table 2) for the 1st (*P* = 0.037), 3rd (*P* = 0.003), and 6th (*P* = 0.032) months after radical prostatectomy. However, this difference eliminated 1 year postoperatively (*P* = 0.579). The results did not change when we examined the contribution of nerve spare in continence rates. Similar results were noticed when we evaluated the severity of incontinence and the impact on quality of life through the ICIQ-SF (Figure 3) and IIQ-SF (Figure 4), respectively. In the first case, significant differences in favor of group B were noticed in the 1st, 3rd, and 6th months, while in the second case these differences were found until the 3rd month after surgery (Table 2). In both cases, there were no statistical differences at the end of the study.

The rates of urge (*P* < 0.001) and stress incontinence (*P* < 0.001) 1 year postoperatively and the rate of stress incontinence 1 month after the operation (*P* < 0.001) were significantly lower in patients who underwent a nerve-sparing radical prostatectomy. However, when we analysed those patients who underwent a nerve spare, patients of group A had significant higher rates of stress incontinence after 1 month (*P* = 0.047) and 1 year (*P* = 0.027) after surgery compared to patients of group B.

## 4. Discussion

Nowadays, an increased number of younger patients are diagnosed with early stage and low-volume prostate cancer, increasing the need for preserving continence and erectile function after radical prostatectomy. In the era of the so-called “trifecta” target and as the postoperative recovery period shortens, much attention is paid to urinary continence after radical prostatectomy [12] since it remains the most feared complication for men, even worse than the erectile dysfunction [9, 13]. As a result, several technical modifications have been attempted in order to achieve better and earlier postoperative outcomes. Actually, the incidence of incontinence at 1 year postoperatively is not very high, and so efforts are being made to achieve an earlier recovery.

As far as the factors that affect the urinary continence postoperatively are concerned, several of them are described in the literature. It has been reported that increasing age, shorter pre- and postoperative urethral length, anastomotic strictures, obesity, low surgeon volume, vesicourethral anastomosis location below the pubic symphysis as seen in postoperative cystography, and previous prostate surgery are negative risk factors for delayed continence recovery and permanent incontinence [14–16]. Several surgical modifications have been described in the literature including the preservation of the urethral rhabdosphincter length, the posterior reconstruction of Denonvilliers' musculofascial plate, preservation of the bladder neck and internal sphincter, bladder neck intussusception, posterior and anterior fixation of the urethra, bladder neck mucosal eversion, preservation of the puboprostatic ligaments, preservation of the endopelvic fascia, and several combinations of all of them [4, 5, 17–21]. Techniques reconstructing the pelvic anatomy have mixed results [6]. Surgical modifications that preserve the natural urinary continence mechanism seem to promote the early recovery of continence [22].

Most of the studies which have been published so far do not exactly refer to the point that urethra can be dissected in order to increase its length without harming the oncological outcome. Based on this gap, we preoperatively defined verumontanum as the proximal limit for urethral dissection. Verumontanum serves as an ideal anatomical landmark easy to recognize during a radical prostatectomy, with no anatomical alterations to be reported so far, while it represents the anatomical limit for preservation of the striated sphincter during transurethral techniques. To our knowledge, this study is the first to describe the potential beneficial role of this surgical modification in both oncological and functional outcomes. An earlier urinary continence recovery was found in patients of group B; however, this difference was eliminated at 1 year postoperatively. Similarly, at the 1st month after surgery, stress and urge incontinence appeared in less patients of group B, while an important number of patients of group A suffered from urgency and nocturia. Concerning the number of pads/day used, there was again a statistically significant difference in favor of group B in the 1st, 3rd, and 6th months postoperatively. In an effort to quantify the differences between the groups and to more precisely estimate the incontinence severity and impact in quality of life, we used ICIQ-SF and IIQ-SF. In both cases, we found important differences in favor of group B for the first postoperative months (Figures 3 and 4). It has to be noticed that there was no statistically significant differences at 1 year postoperatively. Consequently, our surgical modification achieved an earlier continence recovery postoperatively; however, it did not increase the final continence rate after 1 year of follow-up. This fact comes in terms with many other studies reported in the literature, where the incidence of the overall continence did not differ much, compared to the improvement of the early recovery of continence [23].

It is very important that there was no significant difference between the groups concerning the postoperative stage and grade, while the rate of positive surgical margins was not statistically different as well. In addition to the above positive functional outcomes, our technique does not compromise the oncological outcomes. No differences were found in the incidence of extracapsular disease and positive surgical margins (Figure 1), while biochemical relapse at 1 year of follow-up was not statistically different.

The effect of nerve-sparing radical prostatectomy has been proved by several studies [24, 25]. Similar to these results, our study patients had increased rates of postoperative continence with a nerve-sparing technique. However, it is of great importance to notice that, when we made a separate analysis of the patients who underwent a nerve-sparing radical prostatectomy, the rates of postoperative continence were higher in group B patients, proving that maximal urethra preservation has an independent significant role on continence recovery.

The rationale for inducting this surgical modification was based on the fact that, by preserving the verumontanum at the apex of the prostate, we preserve important anatomical structures that pass nearby that area and may play a role in the continence mechanism. By dissecting the urethra more proximally, we kept the autonomic branches that innervate the external sphincter (that pass 3 to 13 mm close to the apex according to Walz et al.) further away from the anastomosis compared to the patients of the first group [26]. Furthermore, by working in a more proximal site away from the external sphincter, the latter is less compromised by the inflammatory process that takes place due to the intraoperative maneuvers at the site of the anastomosis, and consequently an earlier continence recovery may be obtained.

Another explanation that can be tried is based on the idea described in the literature that the largest length of the membranous urethra plays an independent role in having earlier recovery of urinary continence [27, 28]. In our study, by preserving the verumontanum, we preserve the full length of the membranous urethra. Besides, Walz et al. described that the prostate apex may overlap the external sphincter in various ways [26]. Significant overlap may result in much less preservation of the membranous urethra. Schlomm et al. have described a modified surgical technique that preserves the full functional length of the urethra resulting in earlier recovery of continence [29]. Actually, Schlomm et al. describe a technique which reveals the distal limit of the external sphincter from outside to inside by selective ligation of dorsal vein plexus. However, no specific superficial anatomical landmark is described, making the technique demanding and the need for high surgical expertise obligatory. In our study, by using verumontanum as the proximal limit for urethra dissection, we provide an-easy-to assess anatomical landmark for preservation of full length membranous urethra and early continence restoration.

The main limitation of our study is that the tissue underneath the verumontanum is consisting of prostatic tissue. Naturally, there is a possibility for higher rates of positive surgical margins, although in our study this was not the case. Another limitation of the present study is that there are other confounding factors (cardiac disorders, diuretics medication, and new onset sleep apnea) that may affect nocturia and urgency postoperatively and impair the results. Urodynamic definition of overactive bladder and detrusor instability instead of obstructive urethra would be a more reliable way of urgency estimation. Missing follow-up results is another important limitation.

As we reported above, increasing the urethral length may give higher rates of early continence recovery in patients who have undergone a radical prostatectomy. However, the exact limit of urethral dissection is controversial. In an effort to define the most proximal limit of urethral dissection, we used verumontanum to be the one. The results after 1 year of follow-up, regarding postoperative incontinence rates and the time to recover, are very encouraging, while this surgical modification did not affect the oncological outcomes.

## 5. Conclusion

Postoperative incontinence following radical prostatectomy represents a significant complication. Several surgical techniques have been described with the majority of them to preserve the normal pelvic anatomy, in an effort to decrease the incontinence impact and the recovery time. Our surgical modification preserves part of the anatomy of the urinary continence mechanism. The preservation of maximal urethral length until the level of verumontanum showed earlier recovery of continence without compromising the oncological outcome. It has to be noted that it is necessary to deepen our understanding upon the anatomy around the prostate apex and about the pathophysiologic mechanisms of incontinence.

## Figures and Tables

**Table 1:** Study cohort.

*n*	244
Age (years), mean ± std	66.2 ± 6.48
PSA (ng/mL), mean ± std	10.8 ± 9.22
Clinical stage, *n* (%)	
T1c	181 (74.2)
T2a	45 (18.4)
T2b	10 (4.1)
T2c	8 (3.3)
Preoperative Gleason score, *n* (%)	
≤6	123 (50.4)
7	94 (38.5)
≥8	27 (11.1)
Neurovascular spare, *n* (%)	
Yes	141 (57.8)
No	103 (42.2)
Pathological stage, *n* (%)	
T2a	28 (11.5)
T2b	9 (3.7)
T2c	140 (57.4)
T3a	39 (16.0)
T3b	28 (11.5)
Postoperative Gleason score, *n* (%)	
≤6	94 (38.5)
7	110 (45.1)
≥8	40 (16.4)
Surgical margins, *n* (%)	
Positive	44 (18.0)
Negative	200 (82.0)
Biochemical relapse, *n* (%)	
Yes	39 (16.0)
No	205 (84.0)

Std: standard deviation.

**Table 2:** Clinical and pathological findings regarding the preservation of urethra until the level of verumontanum.

	Group A	Group B	*P*
Number of patients	115	129	
Age (years), mean ± std, IQR	66.8 ± 6.68, 9	65.6 ± 6.28, 8	0.058^†^
PSA (ng/mL), mean ± std, IQR	9.15 ± 4.15, 5.7	9.43 ± 5.82, 4.83	0.562^†^
Preoperative Gleason score, *n* (%)			0.969^‡^
≤6	57 (49.6)	66 (51.2)	
7	45 (39.1)	49 (38.0)	
≥8	13 (11.3)	14 (10.9)	
Pathological Gleason score, *n* (%)			0.871^‡^
≤6	45 (39.1)	49 (38.0)	
7	50 (43.5)	60 (46.5)	
≥8	20 (17.4)	20 (15.5)	
Pathological stage, *n* (%)			0.433^‡^
T2a	12 (10.4)	16 (12.4)	
T2b	4 (3.48)	5 (3.88)	
T2c	63 (54.8)	77 (59.7)	
T3a	18 (15.7)	21 (16.3)	
T3b	18 (15.7)	10 (7.75)	
Extracapsular disease, *n* (%)			0.204^‡^
Yes	36 (31.3)	31 (24.0)	
No	79 (68.7)	98 (76.0)	
Surgical margins, *n* (%)			0.562^‡^
Negative	96 (83.5)	104 (80.6)	
Positive	19 (16.5)	25 (19.4)	
Biochemical relapse, *n* (%)			0.321^‡^
Yes	20 (17.4)	19 (14.7)	
No	95 (82.6)	110 (85.3)	
Urgency at 1st month, *n* (%)			<0.001^‡∗^
Yes	71 (61.7)	45 (34.9)	
No	44 (38.3)	84 (65.1)	
Nocturia at 1st month, *n* (%)			<0.001^‡∗^
Yes	74 (64.3)	47 (36.4)	
No	41 (35.7)	82 (63.6)	
Incontinence at 1st month, *n* (%)			
Urge	29 (25.2)	18 (14.0)	0.026^‡∗^
Stress	62 (53.9)	53 (41.1)	0.045^‡∗^
Incontinence at 1st year, *n* (%)			
Urge	12 (10.4)	11 (8.53)	0.611^‡^
Stress	24 (20.9)	23 (17.8)	0.548^‡^
Pads/day, mean ± std, IQR			
1 month	1.27 ± 0.91, 1	1.02 ± 0.81, 1	0.037^†∗^
3 months	0.58 ± 0.74, 1	0.34 ± 0.63, 1	0.003^†∗^
6 months	0.34 ± 0.62, 1	0.18 ± 0.44, 0	0.032^†∗^
12 months	0.11 ± 0.44, 0	0.06 ± 0.27, 0	0.579^†^
ICIQ-SF, mean ± std, IQR			
1 month	7.54 ± 5.42, 9	5.13 ± 4.39, 8	0.001^†∗^
3 months	4.97 ± 6.08, 11	1.96 ± 3.96, 0	<0.001^†∗^
6 months	2.44 ± 4.63, 3	1.23 ± 3.20, 0	0.021^†∗^
12 months	2.57 ± 5.54, 0	0.92 ± 3.31, 0	0.11^†^
IIQ-SF, mean ± std, IQR			
1 month	5.99 ± 4.97, 9	4.46 ± 4.22, 8	0.021^†∗^
3 months	3.48 ± 4.36, 7	1.35 ± 2.70, 0	<0.001^†∗^
6 months	1.56 ± 3.34, 0	0.81 ± 2.51, 0	0.057^†^
12 months	0.63 ± 2.56, 0	0.38 ± 1.62, 0	0.590^†^

^†^Mann-Whitney *U* test, ^‡^Chi-square test, *statistically significant, std: standard deviation, IQR: interquartile range, ICIQ-SF: international consultation on incontinence questionnaire-short form, and IIQ-SF: incontinence impact questionnaire-short form.
